# Burials in Charcoal

**Published:** 1855-09

**Authors:** 


### BURIALS IN CHARCOAL.

The question of interments in charcoal, brought forward in the last number of this Journal, has attracted a degree of attention which we did not expect at the time the article was written. The subject, it seems, is one of practical interest to various classes of society:—to the poor, who are anxious to avoid the expenses of far distant extra-mural burial; to the clergyman, who is interested in the full retention of his burial fees; to many of the wealthy or competent, who have strong and sensitive feelings in favour of special burial places; and especially to the true sanitary and social reformer, who looks with horror on any system that shall give rise to the long retention of the dead in the houses of the living, or to the slow dissolution of organic remains, either within or without our towns and cities.

In the various communications we have received on this subject, several objections and questions have been raised. It has been urged that the cost of charcoal would render its use inapplicable as regards burials, that numerous prejudices against it would stand in its way, and that it might not be so effectual in hastening the destruction of the animal tissues as has been supposed. Regarding the first of these points, we have made very careful inquiries, and find that even at the present moment the outlay of one or two shillings would defray all the extra expense attendant upon a single burial with charcoal. But were the plan we suggest to be extensively adopted, even this trifling expenditure would, owing to the increased importance of charcoal as a commercial article, be considerably reduced; for charcoal is one of those agents which admits of an unlimited supply, and is reapplicable even when it has once been used. On the points connected with the manufacture and commerce of this substance, we refer the reader to the excellent paper by Mr. Durden, of Leeds, published in another part of this number of the JOURNAL OF PUBLIC HEALTH.

In regard to the second objection, viz., that the system proposed would be in opposition to the feelings of the community, we could show a great deal to prove that this objection is not founded on fact. There lies before us at this moment a letter (for the perusal of which we are indebted to the Secretary of the Irish Amelioration Society), in which the writer, an intelligent man belonging to the middle classes, describes that some months ago, having witnessed the effects of charcoal, he spontaneously used it for the burial of his wife, being urged to do so by a deep conviction that he was acting on scientific and sanitary principles. The writer states that he surrounded the remains of the deceased with four inches of peat charcoal, and in this way had her interred. We are further informed, that in more than one rural churchyard near to London where burials in vaults have recently taken place, the coffins of the deceased were imbedded in charcoal. These facts plainly indicate, that the prejudices so much feared are in the main imaginary. Indeed, in this age, the old Egyptian feeling for long preservation of the dead has well nigh disappeared, while science and true humanity alike dictate, that the law of nature which ordains that organic substances from whence vitality has passed, shall, in the end, and in spite of all human attempts to the contrary, be returned to their primitive elements, and replay their parts in the work of the universe, that this law ought neither to be forgotten nor opposed.

Moreover, the plan we propose, and which it seems has already been adopted, imposes no innovation in the solemn rites of burial. In this respect it differs from many other rapid modes of disposing of the dead which are or have been used, such as destruction by fire, or burials in the sea. It demands, indeed, only that a few bushels of charcoal be made, instead of earth, to surround the dead.

In relation to the third question, the power of charcoal in destroying animal matter, and rendering the products of its destruction innocuous, there can be no reasonable doubt.

On this point, however, considerable errors prevail; for the old notion is still held, even by educated people, that charcoal possesses antiseptic or preserving powers. This popular belief, which can easily be excused, seeing that our most eminent chemists have until recently taught it, is one of those absolute fallacies in which even the scientific world sometimes indulges. The simple truth is, that with the exception of fire itself, nothing is so powerfully destructive of dead organic substance as charcoal; for the burial of such substances in charcoal sets up a chemical process, which is nothing more nor less than a slow combustion. As a proof of effects in this respect, we are informed by the Messrs. Turnbull, the well-known charcoal manufacturers in Glasgow, that in their original experiments on this subject they found that when so large an animal as a horse was so buried as to be surrounded by six or eight inches of charcoal, every part except the skeleton was destroyed in the space of twelve months, and without any deleterious emanations. They found, too, that moisture from rain did not materially affect the destructive process. From experiments of the same kind which we have observed on a smaller scale, we are fully prepared to substantiate these statements; and we have the authority of Dr. Stenhouse for stating that peat charcoal would answer for burial purposes, and that the reduction of the substance to a state of fine division is not necessary.

The subject thus laid before the reader is one demanding very earnest attention; for the accomplishment of the object proposed would probably solve a sanitary and social difficulty, which has been felt from the first ages of the world. It is far from our wish to give countenance to the old system of interment upon interment in the midst of cities; a system which it was Mr. Walker's great mission to uproot. But we do wish to be understood as opposing forcibly the extreme ideas for far distant extra-mural interments which are now the order of the day, which inflict a heavy and unnecessary tax on the poor, and which have a moral evil by prolonging the period of interment.

Within accessible distances from our most densely populated districts, such burial grounds might, under scientific supervision, be made as should answer every purpose, sanitary and social. But without urging this point we shall have done some service if we have only succeeded in sustaining the essential idea that there is a simple process of burial which, even in the rural cemetery might be applied with advantage, and which in the common graveyard is absolutely indispensable.

